# Future land-use change predictions using Dyna-Clue to support mosquito-borne disease risk assessment

**DOI:** 10.1007/s10661-023-11394-4

**Published:** 2023-06-07

**Authors:** Miarisoa Rindra Rakotoarinia, Ousmane Seidou, David R. Lapen, Patrick A. Leighton, Nicholas H. Ogden, Antoinette Ludwig

**Affiliations:** 1 Département de Pathologie Et Microbiologie, Faculté de Médecine Vétérinaire, Université de Montréal, 3200 Sicotte, Saint-Hyacinthe, Québec J2S 2M2 Canada; 2 Groupe de Recherche en Épidémiologie Des Zoonoses Et Santé Publique (GREZOSP), Faculté de Médecine Vétérinaire, Université de Montréal, 3200 Sicotte, Saint-Hyacinthe, Québec J2S 2M2 Canada; 3 Department of Civil Engineering, University of Ottawa, 161 Louis Pasteur, Ottawa, ON K1N 6N5 Canada; 4 Ottawa Research and Development Centre, Agriculture and Agri-Food Canada, 960 Carling Ave, Ottawa, ON K1A 0C6 Canada; 5 Public Health Risk Sciences Division, National Microbiology Laboratory, Public Health Agency of Canada, 3190 Sicotte, Saint-Hyacinthe, Québec J2S 2M2 Canada

**Keywords:** Land-use change, Dyna-CLUE, Scenario, Mosquitos, Eastern Ontario

## Abstract

Mosquitoes are known vectors for viral diseases in Canada, and their distribution is driven by climate and land use. Despite that, future land-use changes have not yet been used as a driver in mosquito distribution models in North America. In this paper, we developed land-use change projections designed to address mosquito-borne disease (MBD) prediction in a 38 761 km^2^ area of Eastern Ontario. The landscape in the study area is marked by urbanization and intensive agriculture and hosts a diverse mosquito community. The Dyna-CLUE model was used to project land-use for three time horizons (2030, 2050, and 2070) based on historical trends (from 2014 to 2020) for water, forest, agriculture, and urban land uses. Five scenarios were generated to reflect urbanization, agricultural expansion, and natural areas. An ensemble of thirty simulations per scenario was run to account for land-use conversion uncertainty. The simulation closest to the average map generated was selected to represent the scenario. A concordance matrix generated using map pair analysis showed a good agreement between the simulated 2020 maps and 2020 observed map. By 2050, the most significant changes are predicted to occur mainly in the southeastern region’s rural and forested areas. By 2070, high deforestation is expected in the central west. These results will be integrated into risk models predicting mosquito distribution to study the possibility of humans’ increased exposure risk to MBDs.

**Supplementary Information:**

The online version contains supplementary material available at 10.1007/s10661-023-11394-4.

## Introduction

Over the last few years, projections of the range of pathogens, their reservoirs, and their vectors under global environmental change, have proven fruitful for informing on the epidemiology of different infectious diseases (Altizer et al., 2013; Keeling & Rohani, 2011; Kraemer et al., 2016; Vynnycky & White, 2010). It is possible to predict disease spread according to different scenarios and consider interventions to control disease spread or its impact. This is how, for example, vulnerable patients were identified and targeted for isolation measures during a Zika epidemic in Martinique in 2016 (Cousien et al., 2019).

Determinants of mosquito-borne diseases, one of the main types of vector-borne diseases (VBD), mostly include climate/weather and land use. In Canada, several arboviruses transmitted by mosquitoes have become endemic. Climate is the most studied driver of the spread of these arboviruses geographically (Bartlow et al., 2019; Epstein et al., 1998; Lee et al., 2013; Reiter, 2001). However, the role of other drivers may be relevant and should also be accounted for in the modeling process, especially in projection approaches. Indeed, the elements governing mosquito distribution are multifactorial; they include but are not limited to, aquatic habitat for larvae, the presence of flowers from plants that serve as food for adult mosquitoes, and the presence of vertebrate hosts that supply blood meals for gravid females to permit egg development. By considering multiple drivers of VBD occurrence and spread, more realistic predictions of public health risk can be made (Keeling & Rohani, 2011; Vynnycky & White, 2010), and more efficient disease management actions can be designed.

The effects of either climate or land-use changes on the future geographical distribution of mosquito communities and associated VBD have been addressed independently in previous studies (Altizer et al., 2013; Bowden et al., 2011; Lee et al., 2013; Ogden et al., 2014; Reiter, 2001). However, to our knowledge, few studies have explicitly investigated climate and land-use change implications together.

Climate projections for a region are generally readily accessible and updated. But, this is not the case for simulations of land cover changes for a region of interest. Land-use change models are useful tools that can help project future land-use trajectories helping policymakers interpret causes and potential consequences of land-use dynamics on the target of interest (Verburg & Overmars, 2009; Verburg et al., 2002). The range of disciplines using land-use projection can extend from economics (Sakayarote & Shrestha, 2019; Van der Sluis et al., 2019; Zhang et al., 2013) to ecology (Préau et al., 2019; Trisurat et al., 2010; Waddell et al., 2020).

Each type of land-use modeling approach relies on different techniques (Meiyappan et al., 2014) that vary according to the research goal, geographical area, and data sources and types (Murray-Rust et al., 2014). Agent-based models, cellular automata, artificial neural networks, economics-based models, and Markov chain approaches, for example, have been used in a dynamic land-use change capacity (Schrojenstein Lantman et al., 2011).

In this study, we used dynamic conversion of land-use and its effects (Dyna-CLUE), which generates land-use maps using scenario analysis. Dyna-CLUE is one of the different versions of the CLUE family of models (CLUE, CLUE-s, Dyna-CLUE, and CLUE-Scanner). It is among the most frequently and widely used land-use change models in the past decades (Das et al., 2019; Li et al., 2014; Price et al., 2017; Tizora et al., 2018; Trisurat et al., 2010; Verburg & Overmars, 2009; Verburg et al., 2002). Dyna-CLUE has proven to be broadly useful for decision-making in spatial management activities (Das et al., 2019; Waiyasusri & Wetchayont, 2020).

Our study region, eastern Ontario, Canada, land use is dynamic, and has been undergoing urbanization and intensive agricultural expansion for several decades. Czekajlo et al. (2021) have shown that the average transition rate from natural to urban areas in the greater Ottawa region (eastern Ontario) can be as high as 5.8%. The average rate of change from agricultural to urban areas and from natural to agricultural areas in the greater Ottawa, Ontario, reaches 2.4% and 4%, respectively. Furthermore, the trends of agricultural change in Ontario are unique as it possesses some of the most productive soils in Canada (Smith, 2015), accounting for 25% of Canada’s agricultural production.

Several arboviruses that are transmitted by mosquitoes are known to circulate endemically in Canada, especially in Ontario. West Nile Virus (WNV) was first detected in Ontario in 2002. Since then, Public Health Ontario (PHO) has initiated and maintained surveillance for mosquitoes and mosquito-borne viruses revealing the presence of WNV and other arboviruses such as the Eastern Equine Encephalitis (EEEV), and Californian serogroup viruses (CSV) (Drebot, 2015) including Jamestown Canyon virus (JCV) and Snowshoe Hare virus (SSHV). These arboviruses circulate frequently in southeastern Canada where vector species include *Aedes vexans* and *Culex pipiens-restuans* (the main vectors of WNV), *Culiseta melanura* (the main vector for EEEV), and several potential vectors for CSV (belonging to the genera *Ochlerotatus* and *Culiseta*). There is a high number of mosquito species occurring in this region (67 species in Ontario) (Giordano et al., 2015), and monitoring the evolution of these species in terms of interaction with their habitats (as determined by land use and climate), animal hosts and pathogens, may enhance our capacity to predict risk from arboviruses. With a changing climate and, possibly land-use change, the risk of exposure to arboviruses is likely to increase (Beard et al., 2016; Franklinos et al., 2019; Patz et al., 2008). Warmer temperatures associated with climate change can accelerate mosquito development, biting rates, and reduce the extrinsic incubation of pathogens in the mosquito, while rainfall can impact occurrence of breeding sites for mosquitoes (Beard et al., 2016; Franklinos et al., 2019). Urban, agricultural areas, and natural habitats such as woodlands and wetlands are novel ecosystems with varying capacity to support mosquito populations (Franklinos et al., 2019; Norris, 2004; Ortiz et al., 2022; Rakotoarinia et al., 2022). Attempting to understand how these ecosystems influence composition and abundance of mosquitos is essential to understanding the ecology of mosquito species and predict risk from the diseases they transmit.

This paper presents the first step of a project that aims to model and predict the combined effects of land-use change and climate change on the abundance of *Culex pipiens-restuans*, the main vector of West Nile virus in eastern Canada, and on the diversity of mosquitoes in Eastern Ontario. This first step comprises the development, simulation, and validation of land-use change scenarios and maps that can then be combined with climate projections.

The Dyna-CLUE model used in this study requires two main inputs: historical land-use change trends and spatial suitability equations. Historical land-use maps of the eastern Ontario region from 2014 to 2020 were used to parameterise the model to create possible future land-use trends, according to different scenarios for land-use change, for three future time periods: 2030, 2050, and 2070.

## Methods

### Study region

**Fig. 1:**
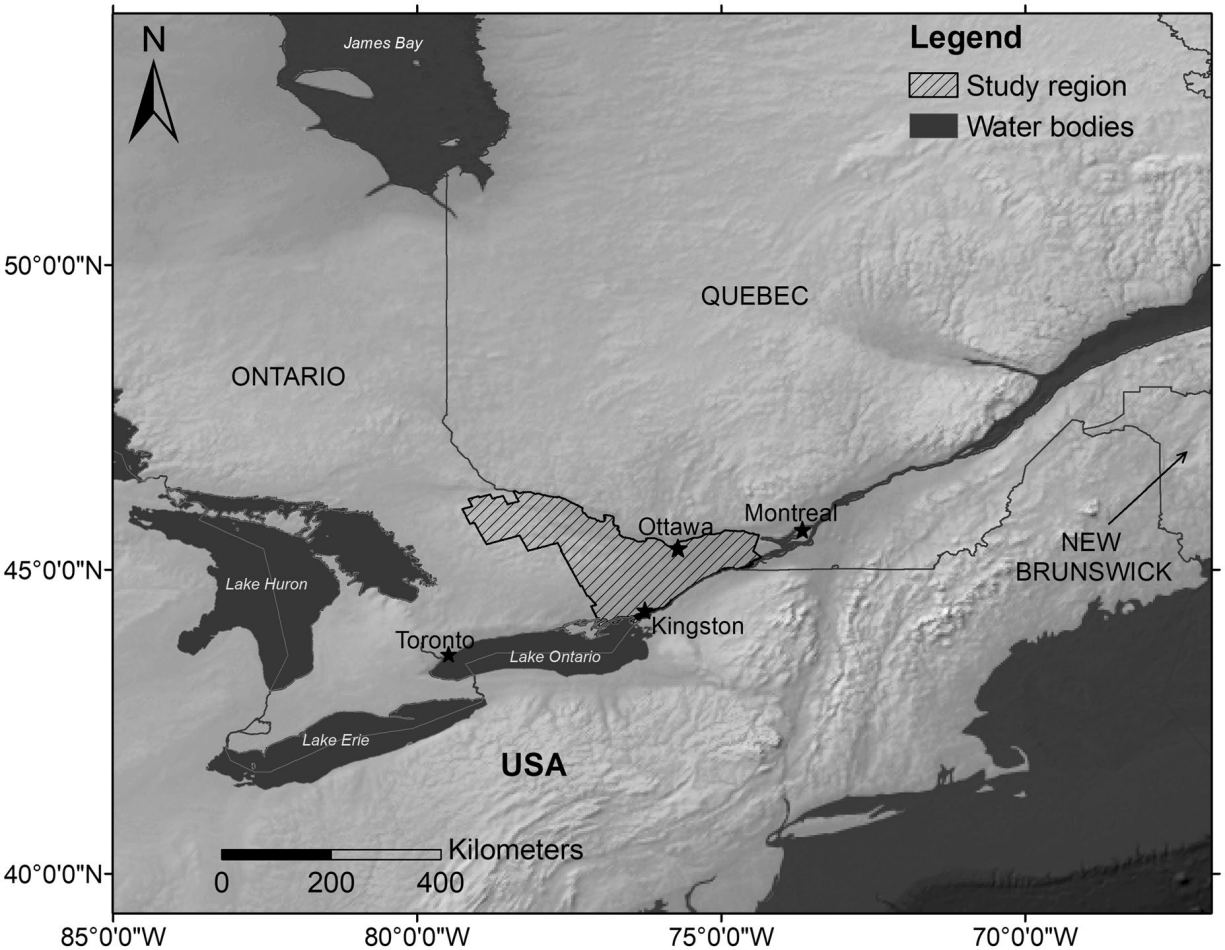
Location of the study region in the eastern region of Ontario province. Made with Natural Earth. Free vector and raster map data @ naturalearthdata.com

The study region (Fig. 1), covering an area of 38 761 km^2^, is located in eastern Ontario, Canada, in a mixed use but primarily agricultural landscape and including 12 counties: Prescott, Glengarry, Stormont, Dundas, Russell, Carleton, Grenville, Lanark, Leeds, Renfrew, Frontenac and Lennox, and Addington. The city of Ottawa is the major urban center in the study area. The eastern part of the study region is predominantly covered by agricultural land uses. Urban areas such as Ottawa and its suburbs are located in the northern areas. The western part is dominated by natural areas (forest and water). According to the Köppen-Geiger climate classification (Kottek et al., 2006), eastern Ontario is characterized by a climate with snowfall, fully humid, and warm summers.

### Modeling approach

**Fig. 2:**
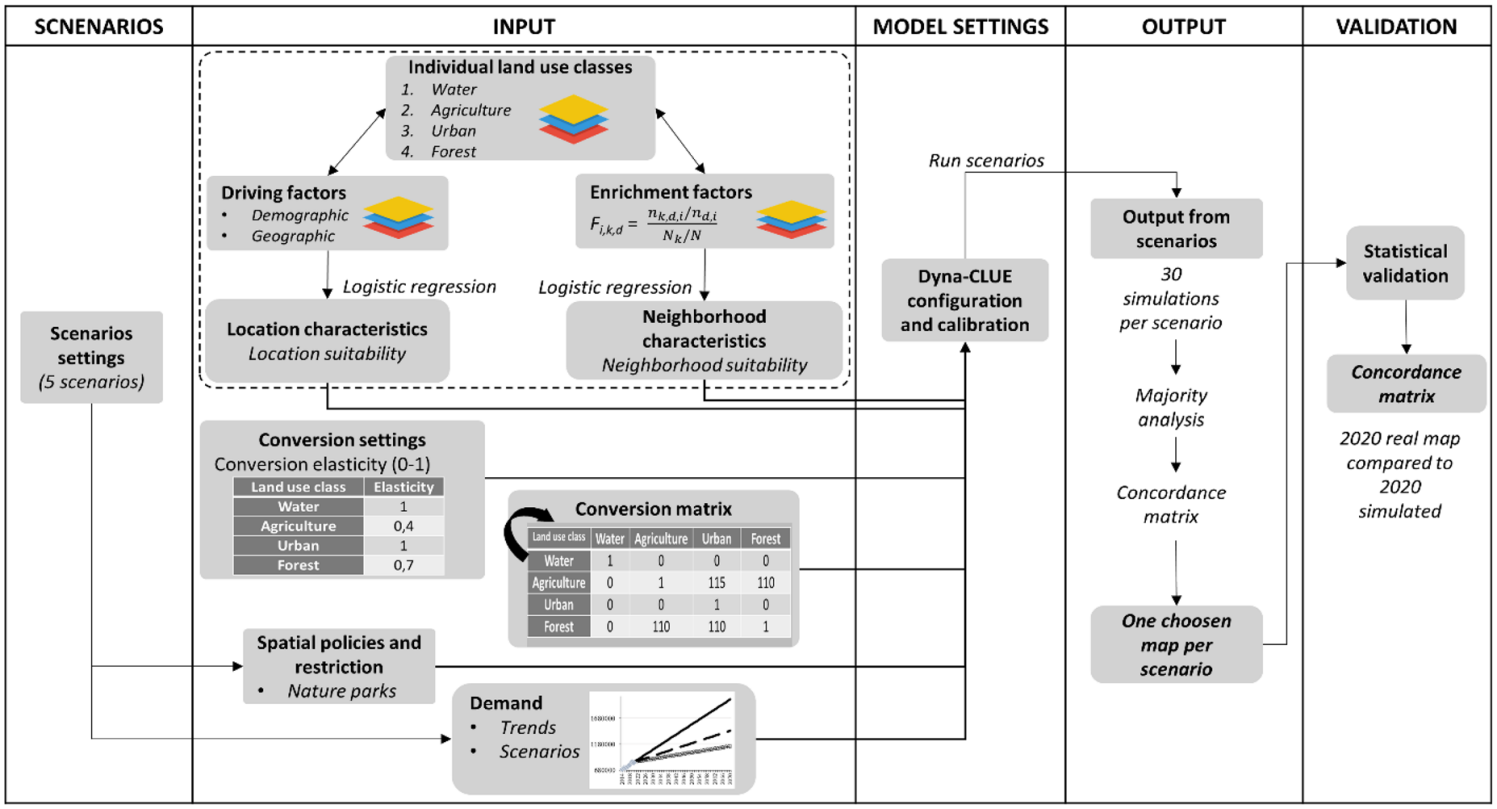
Flowchart of the Dyna-CLUE approach used in this study

Dyna-CLUE is a spatially explicit and scenario-based modeling approach that generates future land-use maps based on historical trends of a given region's existing land-use classes. It integrates demand-driven changes in land area with locally determined conversion processes (Verburg & Overmars, 2009). It comprises a non-spatial module and a spatial module. The non-spatial module calculates the change of areas for all classes of land use from 1 year to another, called the demand. Different approaches can be used to calculate the demand ranging from simple linear trend extrapolations to complex economic models and need to be decided by the user for its specific situation (Verburg et al., 2002). The choice of the method calculation strictly relies on the nature of the most important land-use conversions occurring in the study area and the considered scenarios. In our study, we employed parsimonious linear relationships (linear regression) between the surface areas of the two main land uses that undergo the most change (urban and agriculture) and time (years). The fitted relationships were used to calculate future land-use demand starting from the initial start year (2014). These demands are translated into land-use modification in different areas starting from the initial state year (2014) using the Dyna-Clue spatial module. The spatial module allocates land-use classes on a pixel-by-pixel basis. The allocation process is fully described in (Verburg et al., 2006) and summarized in Fig. 2. The calculation of the historical land-use trends (evolution of the area expressed in hectare corresponding to each land-use class over time) were conducted using logistic regression in R, version 4.0.2 (Team, 2020), the spatial module was carried out in the Dyna-CLUE software, and the simulation output was displayed in ArcMap 10.7.1 (ESRI). The full description of the Dyna-CLUE model is given by (Verburg & Overmars, 2009). The model includes several parameters presented hereafter.

#### Land-use data

Agriculture and Agrifood Canada (AAFC) regularly delivers annual crop inventory maps 30-m spatial resolution (Agriculture & Agri-Food Canada Annual Crop Inventory, 2021). The annual crop inventory data between 2014 and 2020 were extracted from https://www.canada.ca/en/transparency/open.html. The maps were resampled to a 330-m resolution to respect the maximum number of pixels allowed in the Dyna-CLUE model (Verburg & Overmars, 2009; Verburg et al., 2004).

Based on previous studies, we assumed that water, forest, agriculture, and urban land uses were important with respect to mosquito abundance and diversity (Moua et al., 2021; Rakotoarinia et al., 2022). Water is an essential requirement for egg and larval development (Medlock et al., 2018; Silver, 2007); however, the type of optimal breeding sites filled with water is not the same for every mosquito species. Some species prefer natural breeding sites while others prefer artificial ones (Medlock et al., 2018; Silver, 2007). Agricultural development and urbanization are also common drivers of mosquito abundance or presence/absence in literature by favoring, or not, their presence and their development (e.g., deforestation and water management) (Norris, 2004). As an example, *Anopheles quadrimaculatus* and *Culex erraticus* affinity with agricultural wetlands is well established (Botello et al., 2013) and *Culex pipiens* is also often described as more abundant in urban environments (Andreadis et al., 2001; Jacob et al., 2009). Similarly, some species are restricted to forested areas such as *Ochlerotatus triseriatus* (Reiskind et al., 2017). It would have been more relevant to separate “wetland” pixels from “water” given their role in mosquito ecology but unfortunately wetlands are not spatially frequent enough to constitute a distinct class of their own (< 5% of the total surface). Thus, a reclassification was conducted by assigning the original land-use class designations to one of these chosen classes. The correspondence between the original and new classes is represented in Table 1.

**Table 1:** Reclassification of land-use classes given in (Agriculture & Agri-Food Canada Annual Crop Inventory, 2021)

Original land uses (Agriculture & Agri-Food Canada Annual Crop Inventory, 2021)	Reclassified land uses (this study)
Water	Water
Exposed land/Barren	Agriculture
Urban/developed	Urban
Greenhouses	Agriculture
Shrubland	Forest
Wetland	Water
Grassland	Agriculture
Pasture/forages	Agriculture
Barley	Agriculture
Oats	Agriculture
Winter wheat	Agriculture
Spring wheat	Agriculture
Corn	Agriculture
Soybean	Agriculture
Beans	Agriculture
Other crops	Agriculture
Forest (undifferentiated)	Forest
Coniferous	Forest
Broadleaf	Forest
Mixed wood	Forest

#### Conversion rules

The conversion settings are implemented in two forms: the conversion matrix and the elasticity.

##### Conversion matrix

The conversion matrix (Table 2) is a set of conversion rules that allows the maximization of the probability of a grid pixel to be devoted to a given land-use class at a specific location (Verburg & Overmars, 2009; Verburg et al., 2002). It indicates which conversions are possible for each land-use class. Code “0” indicates that the conversion is not allowed for the specific land-use class and code “1” indicates that the conversion is allowed. For example, conversion from agriculture to water is not possible. In contrast, conversion from agriculture to urban is allowed.

**Table 2:** Conversion matrix and time to conversion

Land-use class	Water	Agriculture	Urban	Forest
Water	1	0	0	0
Agriculture	0	1	115	110
Urban	0	0	1	0
Forest	0	110	110	1

Code “1” is sometimes followed by the “time to conversion,” defined as the number of years a given land use must remain stable before it is converted into another class. We assumed that agriculture remains stable for 15 years before any possible conversion to urban (Czekajlo et al., 2021; Smith, 2015) and 10 years before any possible conversion to forest (Smith, 2015). The class forest was assumed to remain stable for 10 years before any possible conversion to agriculture or urban (Czekajlo et al., 2021).

##### Elasticity

The conversion elasticity is related to the resistance of a given land-use class to change (Verburg & Overmars, 2009; Verburg et al., 2002). It measures the cost of conversion of a specific land-use class to another and is applied to the locations where the land-use class is found at time *t*. The land uses with high capital investment, such as urban areas, will be more resistant to conversion (Duranton et al., 2015; Environment and Climate Change Canada, 2021; Nuissl & Siedentop, 2021). Once an area is urban, it usually stays that way, but the existing settlement areas grow outward, encroaching on surrounding areas such as forests, agricultural lands, and other natural areas. These pixel areas will have high elasticity values. Thus, high elasticity values are primarily applied to high-cost land-use classes such as urban areas with residential locations or plantations with permanent crops. In contrast, low values may be applied to grassland, bare and similar land-use classes. The values of the relative elasticity range from 0 (easy conversion) to 1 (irreversible change). It is set to 1, 0.4, 1, and 0.7 for water, agriculture, urban, and forest, respectively. The determination of the elasticity values in this study was based on the implementation of several tests in which different values of elasticities were examined. The values where simulated maps were comparable to reference maps were selected. The selected values were consistent with those from relevant publications (Verburg et al., 2002; Xu et al., 2013).

#### Logistic regression 1: location suitability

The location suitability determines the probability of a specific land-use class occurring at a given location. The conversions are expected to occur at locations with the highest “preference” for the specific land use at that moment in time (Verburg & Overmars, 2009; Verburg et al., 2002). This “preference” is based on a stepwise logistic regression. This analysis associates the probability of occurrence of a given land-use class to geographic and demographic conditions (driving factors). Based on (El-Khoury et al., 2014), seven spatially explicit driving factors were selected, including one demographic variable (average population density) and six geographical variables (distance to the nearest cities, distance to the nearest major cities, distance to the nearest small cities, distance to the nearest major roads, distance to the nearest paved roads, and distance to water). The driving factors were retrieved from different sources (Table 3).

**Table 3:** Data sources from which the driving factors for land-use suitability were derived

Variables		Data	Providers	Geographical cover	Links
*Geographical*	• Distance to the nearest cities • Distance to the nearest major cities • Distance to the nearest small cities	Dissemination areas (DA) of the 2016 census	StatCan	Canada	https://www12.statcan.gc.ca/census-recensement/2016/dp-pd/index-fra.cfm
		Census subdivision of 2018 (CSD)	StatCan	Canada	
	• Distance to the nearest major roads • Distance to the nearest paved roads	Road network of Open Street Map (OSM)	Open Street Map	Canada	http://download.geofabrik.de/north-america/canada/ontario.html
		National Highway System (NHS)	National Highway System	Global	https://ouvert.canada.ca/data/fr/dataset/3d282116-e556-400c-9306-ca1a3cada77f
	Distance to water	Annual crop inventory	Agriculture and Agri-food Canada	Canada	https://www.agr.gc.ca/atlas/apps/metrics/index-en.html?appid=aci-iac
*Socio-economic*	Average population density	Population density of 2018	Socio-economic data and application center (SEDAC), NASA	Global	https://sedac.ciesin.columbia.edu/data/set/gpw-v4-population-density-rev11/data-download

These drivers are illustrated in the Supplemental Fig. 1

The logistic regression equation is:

$$\mathrm{log}\left(\frac{{P}_{loc,i}}{1-{P}_{loc,i}}\right)={\beta }_{0}+{\beta }_{1}{X}_{1,i}+{\beta }_{1}{X}_{2,i}+\dots +{\beta }_{n}{X}_{n,i}$$

where *P*_*i*_ is the probability of a grid pixel (*i*) for the occurrence of the considered land-use *n*, *X*s are the driving factors, and *β*s are the regression coefficient of each driving factor.

#### Logistic regression 2: neighborhood suitability

The spatial neighborhood of the land use in a given pixel can partially influence the conversion of this land use (Verburg & Overmars, 2009; Verburg et al., 2002). If the neighborhood function is enabled in Dyna-CLUE, it places a preferential weighting on pixels within the neighborhood of the land-use class to allocate new pixels to that land-use class. The neighborhood suitability is also estimated by a stepwise logistic regression using the land-use class and a set of location parameters called enrichment factors (*F*) (Verburg et al., 2004). The enrichment factor is defined by the occurrence of a land-use class in the location’s neighborhood relative to the occurrence of this class in the whole study area as follows:

$${F}_{i,kd}=\frac{{n}_{k,d,i}/{n}_{d,i}}{{N}_{k}/N}$$

where

*F*_*i,k,d*_ is the enrichment of neighborhood (*d*) of a location ( *i*) for land-use class (*k*).*n*_*k,d,i*_ is the number of pixels of land-use class (*k*) in the neighborhood with size (*d*) of pixel (*i*)*n*_*d,i*_ is the total number of pixels in the neighborhood

*N*_*k*_ is the number of pixels with land-use class (*k*) in the study area.

*N* is the total number of pixels in the study area.

Similar to the location suitability, the equation defines the logit model corresponding to the neighborhood suitability:

$$\mathrm{log}\left(\frac{{P}_{enr,i}}{1-{P}_{enr,i}}\right)={\beta }_{0}+{\beta }_{1}{F}_{1,i}+{\beta }_{2}{F}_{2,i}+\dots +{\beta }_{n}{F}_{n,i}$$

In this present work, enrichment factors *F*_*i,k,d*_ were calculated for five different distances from the central pixel that were used by El-Khoury et al. (2014); for their study region is part of ours: *d* = 330 m (1 × 1), 660 m (3 × 3), 990 m (5 × 5), 4950 m (29 × 29), and 9900 m (59 × 59), reminding that the size of a pixel is 330 m × 330 m. We have used square rings at the distance *d* from the central grid pixel as neighborhoods as proposed by Verburg et al. (2004).

#### Land-use requirements (demands)

**Fig. 3:**
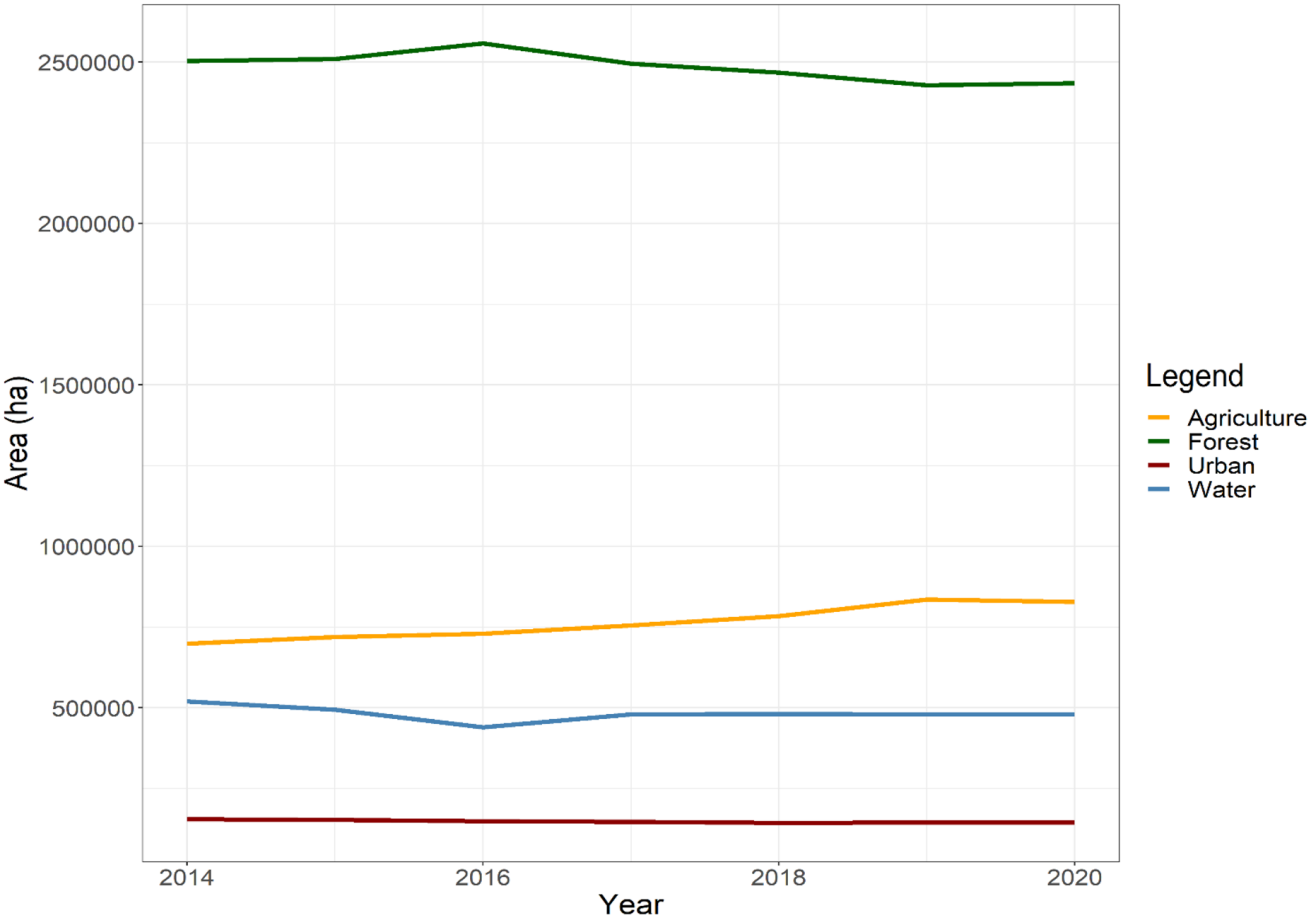
Historical trends between 2014 and 2020 derived from remote sensing data (annual crop inventory) (Agriculture & Agri-Food Canada Annual Crop Inventory, 2021)

Dyna-CLUE projects the evolution of land use based partially on past land-use trends. The calculation of the land-use requirements is based on the extrapolation of land-use change trends from the recent past to the near future for the baseline scenario (see scenario settings section). The time series of the historical land-use maps were generated from the data of the annual crop inventory from 2014 to 2020 (Fig. 3), and the trend was calculated as follows:

- *Agriculture*: a linear trend was estimated for agriculture between 2014 and 2020.
- *Urban*: urban areas vary significantly over time from the observed data. For this reason, the trend related to urban was formulated as an average of the annual number of pixels of this class between 2014 and 2020. A straight horizontal line was considered the baseline trajectory of this class, assuming no urban growth.
- *Forest*: this specific land-use class was estimated as any area which is not agriculture, water, or urban.
- *Water*: similar to the forest class, no trend line was developed for the class “water” as it was assumed that no change would apply to this class. The corresponding areas remain unchanged throughout the study period.

#### Scenarios settings

**Fig. 4:**
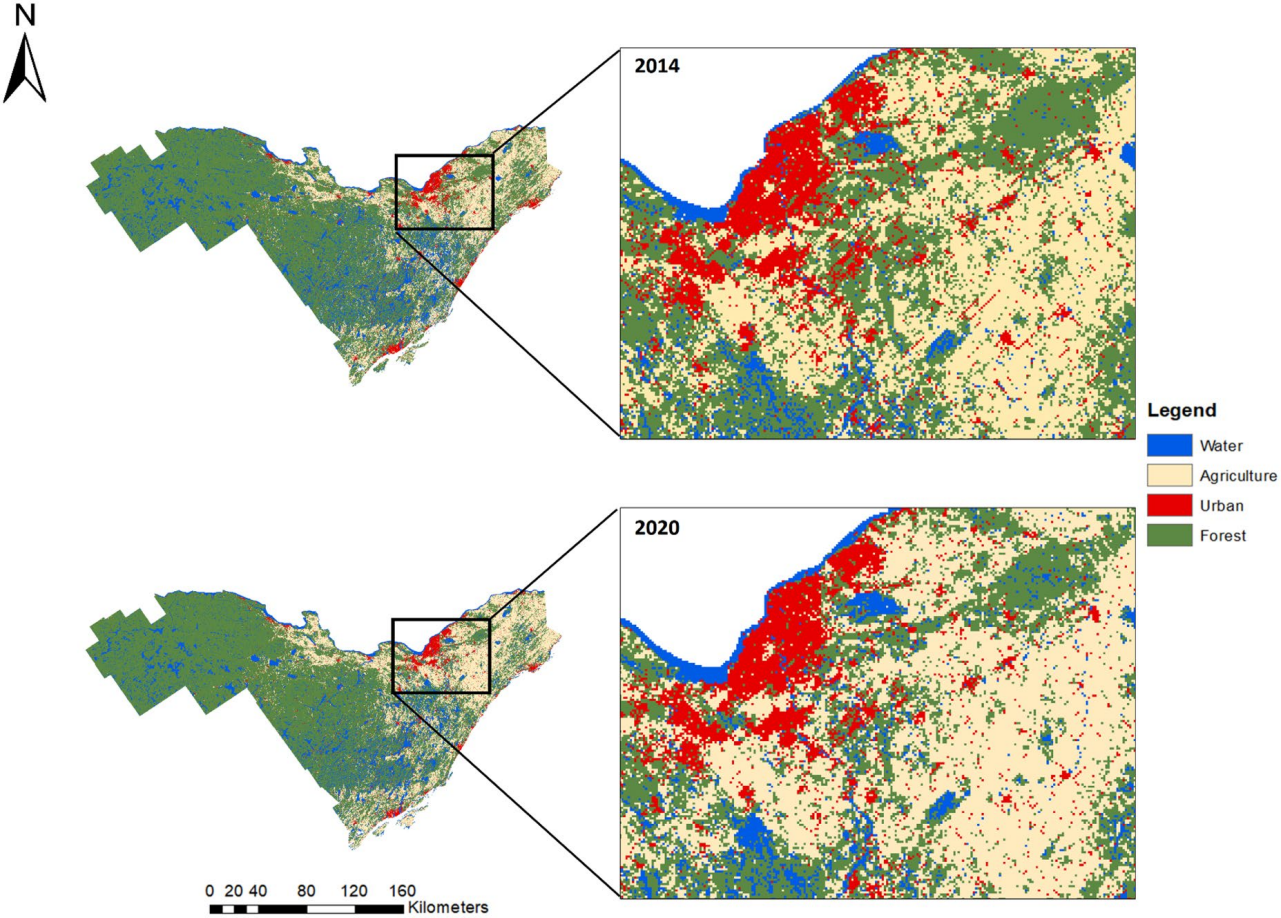
Observed changes between 2014 and 2020 in the eastern Ontario region

Simulations are based on scenarios that need to be considered and decided by the user for the specific context of study (Verburg et al., 2002). To formulate our scenarios, the evolution of all land classes was driven by the urban and the agricultural classes (El-Khoury et al., 2014; Price et al., 2017). First, we used the 2014 map (Fig. 4) as a starting point to simulate a 2020 map to validate our method. Then we used the 2020 map as an initial map to simulate future land uses.

Several scenarios were considered to capture the uncertainty of the trajectories of the abovementioned driving forces. We have established scenarios that assume that the currently observed trend for agriculture will remain unchanged or increase in the future and that the urban areas will remain stable or increase over time. Other scenarios consist of taking into account possible changes in human behavior regarding urban densification, and awareness of the importance of the conservation of natural areas. These scenarios predict little or no future increase in agricultural and urban areas.

**Fig. 5:**
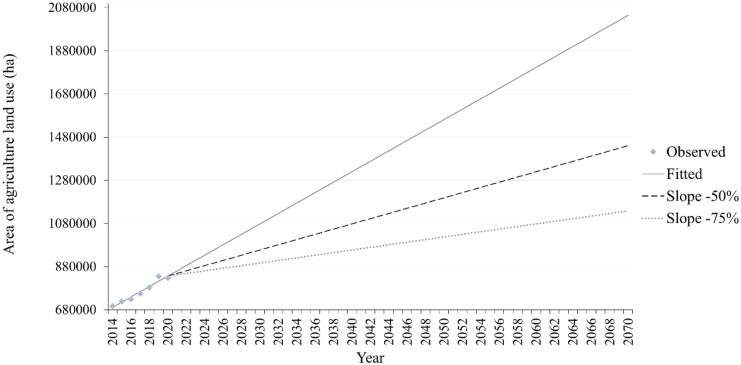
Observed trends and scenarios for the agriculture land-use class

Agricultural scenarios were developed as follows. The trend of observed changes in historical land-use demand, between 2014 and 2020, was obtained by linear regression fitted on the historical values (2014 to 2020) of the given land-use area. The produced linear relationship was used to extrapolate the areas beyond 2020 to 2070. The obtained scenario was considered the base scenario for agriculture. Two additional trajectories of the agricultural area corresponding to a slowdown in agricultural intensification are proposed, one of which foresees a slowdown of 50% and the other of 75% (Fig. 5).

**Fig. 6:**
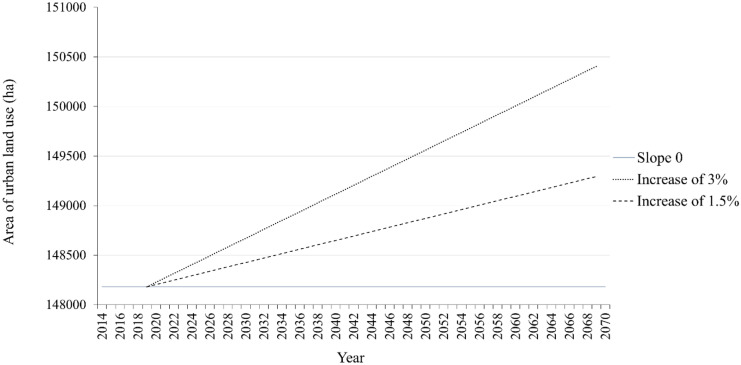
Averaged area of urban area between 2014 and 2020 maps (slope 0) and scenarios (increases of 1.5% and 3%) for the urban land-use class

An average of the total annual pixels over the 7 years of observed data was calculated for the urban class from which a straight line (of slope 0) was drawn, constituting the baseline scenario linked to urban areas. Two other trajectories assuming yearly increases of 1.5 (moderate urban growth) and 3% (strong urban growth) from this horizontal line were proposed (Czekajlo et al., 2021) (Fig. 6).

Five final scenarios were developed based on different combinations of the trajectories predicted for the two classes (urban and agriculture) above to simulate land-use changes in the eastern Ontario region based on the original land-use map of 2014. These scenarios represent different urbanization degrees or agricultural expansion, with accompanying areas that are protected from development. The forest class absorbs the changes resulting from the modifications of the urban and agricultural classes.

##### Scenario 1: Normal development

This first scenario reflects a normal urban and agricultural development, corresponding to a moderate increase of 1.5% in the urban class combined with the base scenario for agricultural growth. In other words, urbanization occurs at a moderate speed, and agricultural practices continue to increase at the current rate without any restriction.

##### Scenario 2: Reduced agriculture expansion

For this scenario, urbanization continues to evolve normally (slight increase), but agricultural growth is slowing down (− 50% from baseline). This scenario assumes measures to limit climate change and conserve natural capital, and perhaps addresses a changing workforce away from farming.

##### Scenario 3: Major urban development A

This scenario is associated with a significant increase in urban development at the expense of agricultural land.

Strong urbanization (3%) but a drastic slowdown in agricultural growth (− 75%) are expected. Urban areas continue to spread at the current rate to meet the population’s needs, mainly at expense of agricultural areas.

##### Scenario 4: Major urban development B

This scenario reflects a major increase in urban development and normal increases in agricultural development at the expense of natural land. High urbanization (3%) and a continuation of agricultural spread at expense of forested areas.

##### Scenario 5: Protection of natural capital

In this scenario, stabilization of urban and agricultural development occurs, assuming a higher ecological awareness and urban intensification vs. extensification. Urban areas stop sprawling, and agricultural growth is strongly reduced (− 75%). This scenario could assume strong measures to limit (mitigate) climate change and reduced extensification of anthropogenic activities.

### Modeling assessment

#### Assessment of the logistic regressions

The goodness of fit of location suitability and the neighborhood equations derived from the stepwise logistic regressions was assessed by the values of ROC (receiver operator characteristics) (Pontius Jr & Schneider, 2001; Smith, 2015). This metric compares the observed values that are binary data over the whole range of predicted values. High area values under the ROC curve (AUC) indicate a good accuracy of the model.

#### Assessment of the land-use model

In order to assess model accuracy, the 2020 simulated maps were compared to the observed 2020 map (also called the observed map). A visual validation was adopted and was confirmed with two statistical indices (the overall accuracy and the Kappa coefficient: Foody, 2006; Tizora et al., 2018; Wang & Sun, 2016; Zhang et al., 2016)). The statistical approach aims to assess how well the simulated maps agreed with the observed map in terms of quantity and location of pixels in each land-use class. High overall accuracy and Kappa coefficient values indicate a good spatial agreement between the observed map and the simulated map. We assumed that the matrices give unbiased statistics related to the relationship between the reference map and the simulated map (Pontius & Millones, 2011). For the scenarios closest to observed for the year 2020, we computed additional performance measures, including errors of omission and commission and Producer’s and User’s accuracy (Pontius et al., 2008). Errors of omission refer to pixels that were omitted from the correct class in the classified map, whereas errors of commission are computed from reviewing the classified pixels for incorrect classifications. The smaller their values are, the better the model fit. Producer’s and User’s accuracy denote the accuracy from the point of view of the map maker and the map user, respectively. The higher their values are, the better the model fit.

#### Model output choice

Given the stochastic component of the Dyna-Clue model occurring in the allocation process, we made the decision to run 30 simulations per scenario, an important consideration not previously conducted. A reference map was created by performing a majority analysis of the 30 simulations per scenario. In the selection of the simulation to be presented in the final results, we performed statistical analyses through a confusion matrix comparing each of the 30 simulations to the reference map resulting from the majority analysis.

## Results

### Modeling

#### Logistic regression 1: location suitability

Results from the suitability logistic stepwise regression are reported in Table 4. This table shows that not all driving factors were included in the regression equations and each factor contributed differently depending on the land-use class. For all land-use classes, almost all driving factors were important. Population density, distance to major roads, and distance to paved roads were positively correlated with agriculture. In contrast, areas next to cities were identified as presence determinants for agriculture. The population density positively influenced the urban class, and its area increased next to major roads and small cities. However, it was negatively related to distance to small cities and major roads. The spatial distributions of the five land-use classes were best explained by the selected demographic and geographic driving factors as indicated by the ROC (receiver operator characteristics) values. High, moderate, and low values of the area under the ROC curve (AUC) were found for agriculture (0.95), urban land (0.79), and forest (0.69).

**Table 4:** Logistic regression coefficients and intercepts for the location suitability models for agriculture, urban, and forest land-use classes

Driving factors	Agriculture	Urban	Forest
(Intercept)	− 1.36400000	− 4.18500000	− 0.52050000
Density	0.00011690	0.00042490	0.00003231
Distance to nearest cities	-0.00002717	0.00234400	− 0.00012710
Distance to major cities	− 0.00008161	0.00003434	− 0.00001661
Distance to small cities	− 0.00004075	− 0.00239100	0.00016430
Distance to major roads	0.00010500	− 0.00002077	0.00003592
Distance to paved roads	0.00125800	0.00015580	− 0.00011440
Distance to water		0.00087330	0.00229600
ROC values	0.95	0.79	0.69

#### Logistic regression 2: neighborhood suitability

Neighboring effects showed that each class of land use significantly influences the suitability of its neighborhood for water, agriculture, urban, and forest classes for at least one neighborhood size (Table 5). The presence of water in the neighborhood (330 m) is an explanatory factor for each of the other classes. For example, the presence of water at less than 330 m strongly increases the likelihood of having forests compared to the likelihood of having agriculture or urban land uses. ROC values presented in Table 5 also showed that the models were reasonable (ROC > 0.9). Based on these values, the prediction was satisfactory for all land-use classes.

**Table 5:** Logistic regression coefficients and intercepts for the neighborhood suitability equation for agriculture, urban, and wooded land-use classes

Suitability factors	Water	Agriculture	Urban	Forest
(Intercept)	− 5.506903	− 5.253116	− 6.37608	− 5.697454
Water 330 m	1.097180	0.145411	0.25576	0.405746
Agriculture 330 m	0.286562	1.786378	0.67688	0.626255
Urban 330 m	0.070242	0.094928	0.30187	0.100921
Forest 330 m	1.436741	1.027627	1.17628	5.328474
ROC values	0.93	0.97	0.98	0.95

#### Land-use changes simulations according to the five scenarios (2030, 2050, and 2070)

**Fig. 7:**
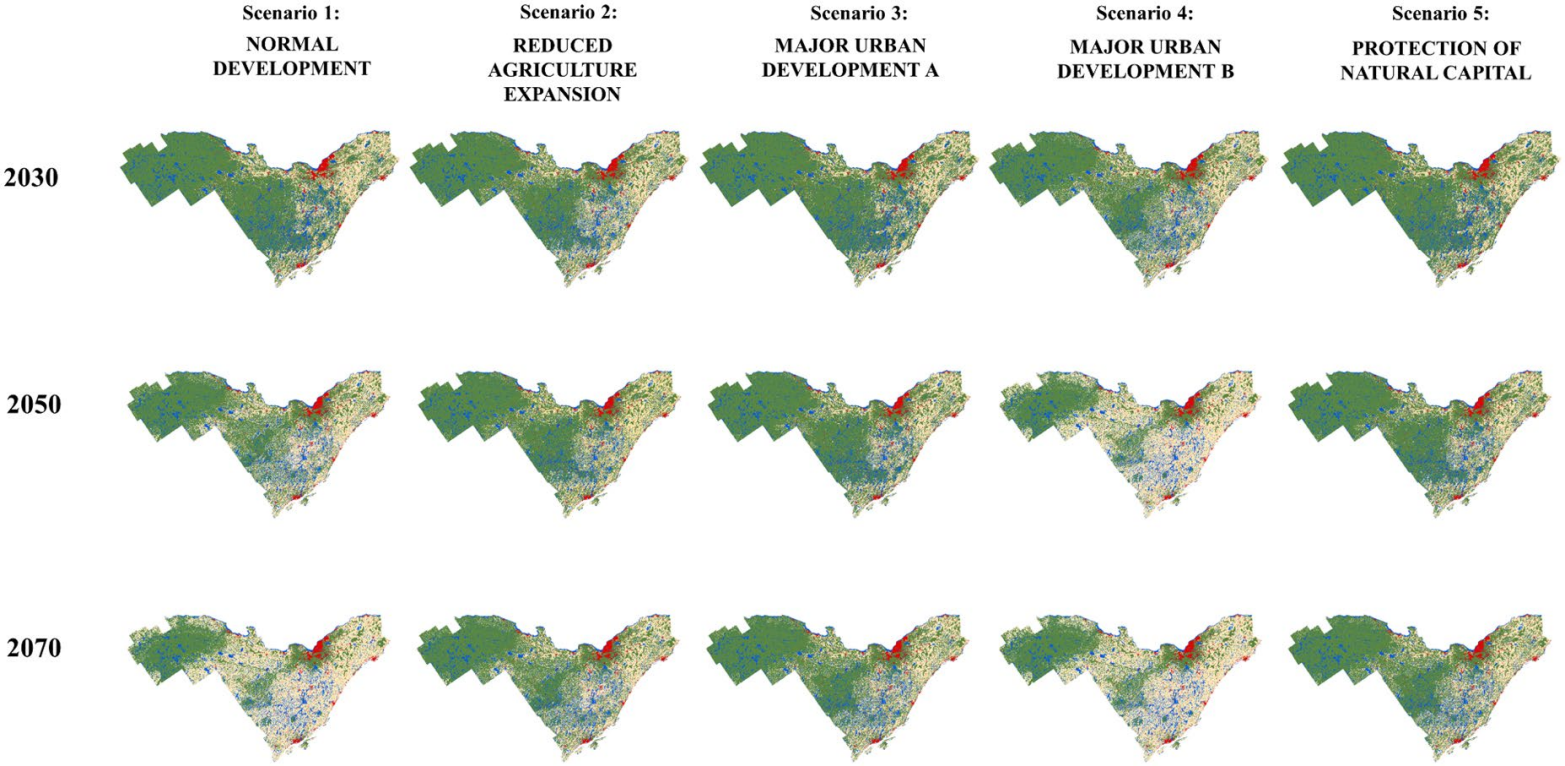
Land-use patterns in 2030, 2050, and 2070 simulated by the Dyna-CLUE model for eastern Ontario, Canada. Normal development: urbanization continues at a moderate rate, and agricultural practices continue to increase at the current rate. Reduced agriculture expansion: this scenario assumes a slowdown in urbanization and agricultural practices. Major urban development A: urban areas continue to spread at the current rate at expense of agricultural areas. Major urban development B: urban areas continue to spread out as well as agricultural areas to the detriment of forest areas. Protection of natural capital: urbanization becomes stable although the population continues to increase. Agricultural practices continue to increase but in a moderate way

Simulated maps for 2030, 2050, and 2070 for each of the five scenarios are displayed in Fig. 7. Compared to the observed map of 2020, the simulated maps of 2030 for all five scenarios did not display substantial changes except for scenarios 2 and 4 (reduced agriculture expansion and major urban development B). Changes became more visible from the year 2050 and after for all scenarios. For three scenarios (reduced agriculture expansion, major urban development A, and the protection of natural capital), the results do not reveal apparent changes in the patterns of each land-use class, especially for 2030 and 2050. In 2070, there will be a moderate expansion of agricultural areas at the expense of the forested regions for these three scenarios. Compared to normal development and the major urban development B scenarios, these three scenarios assumed less demand for agriculture leading to higher remaining forested land and natural areas in the coming years.

The results of the normal development (scenario 1, current agricultural growth rate and normal urban development with an increase of 1.5%) and the major urban development B (scenario 4, urban and agricultural spread to the detriment of forested areas) scenarios showed different land-use patterns, especially in the years 2050 and 2070. A more pronounced and progressive loss of wooded areas was observed in the central west of the study region, which were mostly converted into agricultural lands between 2030 and 2070. In 2070, the patch of forest has substantially decreased by 50% for both scenarios. These two scenarios also saw the most significant increase in the agricultural class in 2070 reaching 147% (Table 6). An increase of urban areas around 2070 demonstrates the strong influence of urban neighborhoods. However, agricultural areas expand rapidly in the major development B (scenario 4, urban and agricultural spread to the detriment of forested areas), resulting in a more substantive forest loss. In contrast, around 2070, the deforestation trend around greater Ottawa is expected to reverse. Forested areas are predicted to appear around the city of Ottawa at expense of agriculture for both scenarios (scenarios 1 and 4). None of the five scenarios has shown a substantial change in the urban areas (Table 6), even for scenarios that assumed higher amounts of urban class increase. Thus, the total urban area remained quite stable, and the percentage of change was comparable between the five scenarios (an increase of 102.2%).

**Table 6:** Land-use changes across scenarios for 2070 (resulting from the majority analysis) and percentage of change compared to the 2020 observed map

Scenarios	Agriculture (ha)	Urban (ha)	Forest (ha)
2020 real map	828,195.39	144,989.46	2,434,503.06
Normal development	2,044,727.63 (+ 146.89%)	149,293.97 (+ 102.97%)	1,200,166.23 (− 50.7%)
Reduced agriculture expansion	1,440,705.09 (+ 73.96%)	149,293.97 (+ 102.97%)	1,804,188.78 (− 25.89%)
Major urban development A	1,138,693.81 (+ 37.49%)	150,405.34 (+ 102.74%)	2,105,088.68 (− 13.53%)
Major urban development B	2,044,727.63 (+ 146.89%)	150,405.34 (+ 102.74%)	1,199,054.86 (− 50.75%)
Protection of natural capital	1,138,693.81 (+ 29.38%)	148,182.60 (+ 102.2%)	2,107,311.42 (− 13.44%)

Enlargements of Fig. 7 for different parts of the study area are shown in Supplemental Fig. 2.

### Modeling assessment

The statistical comparison derived from the pixel-to-pixel confusion matrix of the simulated maps of 2020 and the observed map of the same year reference map suggests a relative agreement between the two sets of maps (Table 7). The accuracy was higher for normal development with a Kappa coefficient of 0.98, reflecting the continuation of the historical trend observed from 2014 to 2020. The other scenarios showed lower accuracy values as they assumed different trajectories of land use compared to the observed trend. The most insufficient accuracy corresponds to the reduced agriculture expansion scenario (Kappa coefficient = 0.372). The Kappa coefficient was similar for the three remaining scenarios (major urban developments A and B, and protection of natural capital) with a value of 0.51.

**Table 7:** Overall accuracy, Kappa coefficient, and other accuracy statistics of the simulated maps of the five scenarios

Scenarios	Statistics	*Minimum*	*Median*	*Average*	*Standard deviation*	*Maximum*
Normal development	*Overall accuracy*	0.9906	0.9909	0.9909	0.0001	0.9912
	*Kappa coefficient*	0.9827	0.9831	0.9831	0.0002	0.9837
Reduced agriculture expansion	*Overall accuracy*	0.6439	0.7123	0.7015	0.0238	0.7244
	*Kappa coefficient*	0.3720	0.4590	0.4460	0.0301	0.4784
Major urban development A	*Overall accuracy*	0.7379	0.7381	0.7381	0.0001	0.7384
	*Kappa coefficient*	0.5100	0.5105	0.5105	0.0003	0.5111
Major urban development B	*Overall accuracy*	0.7377	0.7381	0.7381	0.0001	0.7383
	*Kappa coefficient*	0.5097	0.5105	0.5105	0.0003	0.5110
Protection of natural capital	*Overall accuracy*	0.7379	0.7380	0.7380	0.0001	0.7384
	*Kappa coefficient*	0.5100	0.5104	0.5104	0.0003	0.5111

The normal development scenario is then considered the baseline scenario reflecting the continuation of the observed trends. As such, we computed additional statistics (error of omission, error of commission, User’s and Producer’s accuracy) resulting from the 30 simulations to denote the spatial accuracy (Table 8). Omission and commission errors were less than 4%. Only 4% of the 2020 real map agriculture class was omitted from the classified map, and 1% of the urban class was incorrectly classified. Moreover, the User’s and Producer’s accuracies for all classes were more than 99%, indicating a very good model simulation.

**Table 8:** Accuracy assessment results of the 2020 simulated map corresponding to the normal development scenario

Class	Ground truth (ha)	Simulated (ha)	Omission	Commission	User’s accuracy	Producer’s accuracy
Water	5,270,034	5,270,034	0	0	1	1
Agriculture	8,793,041.77	9,186,683	0.04	0	1	0.96
Forest	27,405,197.23	27,011,556	0	0.01	0.99	1
Urban	1,599,257	1,599,257	0	0	1	1

## Discussion

This study resulted in projected land-use maps for the Eastern Ontario region to year 2070, that differed according to scenarios for land-use change. These results provide possible future land-use projections, but, of course they should be considered with caution particularly in the context of socio-economic and/or political upheavals (Bucała-Hrabia, 2017; Price et al., 2017), such as we have seen recently (e.g., the COVID-19 pandemic, war in Ukraine). Different scenarios provided snapshots of possible changes under more extreme and less extreme drivers of land-use change.

The more extreme scenarios (normal development and major urban development B) mainly predict an extension of the agricultural zones into the forest zones in the center of our study area, stretching towards the west of the study area. Whereas for the less extreme scenarios, changes will occur, but they are less pronounced and will be more visible in 2070. Extensification of agriculture is also predicted, but it will be concentrated in the central areas of the study area. The western forested areas will remain intact. In all the types of scenarios established in this study, the evolution of the urban class remains relatively stable, even assuming increases of this class in certain demand. Urban sprawl is expected, especially in 2070, but this sprawl remains condensed around existing cities. We suspect that this small change in urban areas is due to the low slope linked to urban growth compared to the agricultural growth scenarios. However, the territories occupied by urban areas are a minority in comparison with agricultural areas. To balance this observation, predictions related to the expansion of the urban environment may be less reliable than those of other land-use types because of the assumptions made about the historical trend of the “urban” class. As a reminder, urban areas varied significantly over time from the observed data due to possible bias (ex. change of rules for assigning urban pixels in the annual crop inventories). And we made the decision to use the average value of the number of urban pixels as the historical trend. It would be important to consider other data (such as aerial photography) to improve predicting of the urban environment trend as this type of land use is known to be favorable for certain species of mosquitoes of major interest for public health such as *Culex pipiens-restuans* and *Aedes albopictus*. To our knowledge, there are no data of better quality than those of the annual crop inventories and which are available over a reasonable historical period to serve as input to the Dyna-Clue model.

In this study, the limited use of historical data was due to attempts to maintain consistency in data sources and disposition. We acknowledge that artificial land-use trends can result from use of different data of differing sources, resolution and types when determining historical trends. Data from annual crop inventories provide some reliability in the classification of agricultural pixels, and the study area is predominantly agricultural. This is, therefore, important since the “agricultural” class represents an important determinant for mosquitoes. Yet it is known, for example, that *Anopheles quadrimaculatus*, *Culex erraticus* and *Psorophora columbiae* are associated with temporary wetlands present in agricultural environments (Botello et al., 2013; Kovach & Kilpatrick, 2018), and that *Culex tarsalis* and *C. quinquefasciatus* can breed in agricultural habitats (such as rice fields) (Chuang et al., 2011; Kovach & Kilpatrick, 2018). Thus, even if we had moderate confidence in the classification of urban pixels, the data sources we used remain relevant.

The links that we advance between land-use projections and the abundance and diversity of mosquitoes remain speculative in the context of this work. For more details on this subject, it will be necessary to refer to the next stage of this study which consists of modeling and predicting the combined effects of land-use change and climate change on the abundance of *Culex pipiens-restuans* and the diversity of mosquitoes in Eastern Ontario. Many studies have studied habitat preferences of *Culex pipiens-restuans* and results are not converging. However, deforested areas appear to be positively associated with this species in some studies (Gardner et al., 2014; Rakotoarinia et al., 2022). For this reason, we can expect in this future work that the more extreme scenarios (normal development and major urban development) are likely to increase the numbers of these mosquito species as some mosquito species are more favored in deforested areas, and in agricultural areas. As discussed a priori, agricultural areas can also offer conditions conducive to the development of other mosquito species (presence of irrigation, surrounding wetlands) and can potentially serve as major producers of mosquito vectors of WNV, for example, *C. tarsalis*. This study underlines the relevance of focusing more on these types of landscapes in order to better understand their influence on mosquitoes and the diseases they transmit.

In order to respect the software constraints, the spatial resolution of model input/output was 330 m. Although the spatial resolution of 330 m would not adequately simulate changes in mosquito “micro-habitats” (Okogun et al., 2003; Roberts & Irving-Bell, 1997), the home range of certain mosquito species such as *Culex pipiens-restuans*, for example, is 1 km (Verdonschot & Besse-Lototskaya, 2014). Hence, we did not consider this 330-m resolution a considerable constraint overall.

It is also important to note that the models we used performed well, ensuring their applicability to other research agendas. The assessment of the agreements for specific land-use classes between simulated land-use maps and observed maps, measures of accuracy, reveal a good agreement. Although the choice of drivers could be more complicated (El-Khoury et al., 2014), the results of the suitability modeling suggest that the demographic (population density) and geographical (distances to cities, to roads, and to water) driving factors have good explanatory power for the presence of agriculture, urban, and forested areas.

An additional novelty of our approach is the use of a probabilistic approach in the land-use projections. The land-use projection algorithm in Dyna-CLUE is partly probabilistic as some parameters are sampled in a range provided by the user, meaning that two runs from the same initial map will lead to projections that are slightly different. To account for the randomness in the return, 30 projections were generated and a majority analysis was used to convert the ensemble of projections into a representative map for each time horizon. We are not aware of a land-use projection study which used a similar approach.

## Conclusion

In this study, we set five land-use demand scenarios to project future patterns of water, agriculture, urban, and forestland covers (2030, 2050, and 2070) in eastern Ontario, Canada. The suitability and the neighborhood characteristics were estimated by using stepwise logistic regression. The methodology was validated by statistical comparison between simulated maps and reference maps through a concordance matrix. By applying a new approach to control the uncertainties generated by the random component of the Dyna-CLUE model, which consists of generating several simulations, we could choose maps that adequately illustrate each scenario for future predictions. Analysis of land-use changes in eastern Ontario has reflected two major land conversions: agriculture and urbanization. Results show that the normal development scenario (scenario 1) and the major urban development B (scenario 4) scenarios will allow forest cover loss and agricultural extensification, especially in long term unless strict control measures are undertaken. The results also suggest that the natural environments, including mainly forest areas, can be preserved in the next 30 years if the reduced agriculture expansion (scenario 2), the major urban development A (scenario 3), or the protection of natural capital (scenario 4) occur. Our study has allowed the visualization of different evolutions of agriculture and urbanization in the future which progress by favoring deforestation. The output of this study may thus be used in various contexts where it will be possible to evaluate their consequence on environmental, animal, and public health endpoints. It can also be combined with climate change scenarios. This is the approach that will be taken in our next step to predict the future of both the abundance of the main vector for West Nile virus and the diversity of mosquito.

## Supplementary Information

Below is the link to the electronic supplementary material.

Supplementary file1 (PDF 2993 KB)

## Data Availability

The datasets used in this study are free of access. The sources of these data are given in the method section.

## Declarations

All authors have read, understood, and have complied as applicable with the statement on “Ethical responsibilities of Authors” as found in the Instructions for Authors and are aware that with minor exceptions, no changes can be made to authorship once the paper is submitted.

## References

[CR1] Agriculture and Agri-Food Canada Annual Crop Inventory. (2021). *Annual SpaceBased Crop Inventory for Canada, 2009-2021, Agroclimate, Geomatics and Earth Observation Division, Science and Technology Branch. *Retrieved April 19, 2022 fromhttps://open.canada.ca/data/en/dataset/ba2645d5-4458-414d-b196-6303ac06c1c9

[CR2] Altizer S, Ostfeld RS, Johnson PT, Kutz S, Harvell CD (2013). Climate change and infectious diseases: From evidence to a predictive framework. Science.

[CR3] Andreadis TG, Anderson JF, Vossbrinck CR (2001). Mosquito surveillance for West Nile virus in Connecticut, 2000: Isolation from Culex pipiens, Cx. restuans, Cx. salinarius, and Culiseta melanura. Emerging Infectious Diseases.

[CR4] Bartlow AW, Manore C, Xu C, Kaufeld KA, Del Valle S, Ziemann A, Fairchild G, Fair JM (2019). Forecasting zoonotic infectious disease response to climate change: Mosquito vectors and a changing environment. Veterinary Sciences.

[CR5] Beard, C. B., Eisen, R. J., Barker, C., Garofalo, J., Hahn, M., Hayden, M., Monaghan, A., Ogden, N., & Schramm, P. (2016). *Ch. 5: Vectorborne diseases*. Retrieved May 5, 2022 from https://health2016.globalchange.gov/

[CR6] Botello G, Golladay S, Covich A, Blackmore M (2013). Immature mosquitoes in agricultural wetlands of the coastal plain of Georgia, USA: Effects of landscape and environmental habitat characteristics. Ecological Indicators.

[CR7] Bowden SE, Magori K, Drake JM (2011). Regional differences in the association between land cover and West Nile virus disease incidence in humans in the United States. The American Journal of Tropical Medicine Hygiene.

[CR8] Bucała-Hrabia A (2017). Long-term impact of socio-economic changes on agricultural land use in the Polish Carpathians. Land Use Policy.

[CR9] Chuang T-W, Hildreth MB, Vanroekel DL, Wimberly MC (2011). Weather and land cover influences on mosquito populations in Sioux Falls, South Dakota. Journal of Medical Entomology.

[CR10] Cousien A, Abel S, Monthieux A, Andronico A, Calmont I, Cervantes M, Césaire R, Gallian P, De Lamballerie X, Laouénan C (2019). Assessing Zika virus transmission within households during an outbreak in Martinique, 2015–2016. American Journal of Epidemiology.

[CR11] Czekajlo A, Coops NC, Wulder MA, Hermosilla T, White JC, van den Bosch M (2021). Mapping dynamic peri-urban land use transitions across Canada using Landsat time series: Spatial and temporal trends and associations with socio-demographic factors. Computers, Environment Urban Systems.

[CR12] Das P, Behera M, Pal S, Chowdary V, Behera P, Singh T (2019). Studying land use dynamics using decadal satellite images and Dyna-CLUE model in the Mahanadi River basin. India. Environmental Monitoring.

[CR13] Drebot, M. (2015). Vector-borne diseases in Canada: emerging mosquito-borne bunyaviruses in Canada. *Canada Communicable Disease Report, 41*(6), 117. 10.14745/ccdr.v41i06a01 10.14745/ccdr.v41i06a01 PMC5864308 29769943

[CR14] Duranton G, Henderson V, Strange W (2015). Handbook of regional and urban economics.

[CR15] El-Khoury A, Seidou O, Lapen DR, Sunohara M, Zhenyang Q, Mohammadian M, Daneshfar B (2014). Prediction of land-use conversions for use in watershed-scale hydrological modeling: A Canadian case study. The Canadian Geographer/le Géographe Canadien.

[CR16] Environment and Climate Change Canada. (2021). *Canadian environmental sustainability indicators: Land-use change*. Retrieved June 6, 2022 from https://www.canada.ca/en/environment-climate-change/services/environmental-indicators/land-use-change.html

[CR17] Epstein PR, Diaz HF, Elias S, Grabherr G, Graham NE, Martens WJ, MosIey-Thompson E, Susskind J (1998). Biological and physical signs of climate change: Focus on mosquito-borne diseases. Bulletin of the American Meteorological Society.

[CR18] Foody GM (2006). What is the difference between two maps? A remote senser’s view. Journal of Geographical Systems.

[CR19] Franklinos LH, Jones KE, Redding DW, Abubakar I (2019). The effect of global change on mosquito-borne disease. The Lancet Infectious Diseases.

[CR20] Gardner AM, Lampman RL, Muturi EJ (2014). Land use patterns and the risk of West Nile virus transmission in central Illinois. Vector-Borne Zoonotic Diseases.

[CR21] Giordano BV, Gasparotto A, Hunter FF (2015). A checklist of the 67 mosquito species of Ontario, Canada. Journal of the American Mosquito Control Association.

[CR22] Jacob BG, Lampman RL, Ward MP, Muturi EJ, Morris JA, Caamano EX, Novak RJ (2009). Geospatial variability in the egg raft distribution and abundance of Culex pipiens and Culex restuans in Urbana-Champaign, Illinois. International Journal of Remote Sensing.

[CR23] Keeling MJ, Rohani P (2011). Modeling infectious diseases in humans and animals. Princeton University Press.

[CR24] Kottek M, Grieser J, Beck C, Rudolf B, Rubel F (2006). World map of the Köppen-Geiger climate classification updated. Meteorologische Zeitschrift.

[CR25] Kovach TJ, Kilpatrick AM (2018). Increased human incidence of West Nile virus disease near rice fields in California but not in Southern United States. The American Journal of Tropical Medicine and Hygiene.

[CR26] Kraemer MU, Hay SI, Pigott DM, Smith DL, Wint GW, Golding N (2016). Progress and challenges in infectious disease cartography. Trends in Parasitology.

[CR27] Lee SH, Nam KW, Jeong JY, Yoo SJ, Koh Y-S, Lee S, Heo ST, Seong S-Y, Lee KH (2013). The effects of climate change and globalization on mosquito vectors: Evidence from Jeju Island, South Korea on the potential for Asian tiger mosquito (Aedes albopictus) influxes and survival from Vietnam rather than Japan. PLoS One.

[CR28] Li W, Wu C, Zang S (2014). Modeling urban land use conversion of Daqing City, China: A comparative analysis of “top-down” and “bottom-up” approaches. Stochastic Environmental Research and Risk Assessment.

[CR29] Medlock, J., Balenghien, T., Alten, B., Versteirt, V., & Schaffner, F. (2018). Field sampling methods for mosquitoes, sandflies, biting midges and ticks: VectorNet project 2014–2018. *15*(6), 1435E. 10.2903/sp.efsa.2018.EN-1435

[CR30] Meiyappan P, Dalton M, O’Neill BC, Jain AK (2014). Spatial modeling of agricultural land use change at global scale. Ecological Modelling.

[CR31] Moua, Y., Kotchi, S. O., Ludwig, A., & Brazeau, S. J. R. S. (2021). Mapping the habitat suitability of West Nile virus vectors in Southern Quebec and Eastern Ontario, Canada, with species distribution modeling and satellite earth observation data. *13*(9), 1637. 10.3390/rs13091637

[CR32] Murray-Rust D, Robinson DT, Guillem E, Karali E, Rounsevell M (2014). An open framework for agent based modelling of agricultural land use change. Environmental Modelling Software.

[CR33] Norris DE (2004). Mosquito-borne diseases as a consequence of land use change. EcoHealth.

[CR34] Nuissl, H., & Siedentop, S. (2021). Urbanisation and land use change. In *Sustainable Land Management in a European Context* (pp. 75–99). Springer, Cham. 10.1007/978-3-030-50841-8_5

[CR35] Ogden NH, Milka R, Caminade C, Gachon P (2014). Recent and projected future climatic suitability of North America for the Asian tiger mosquito Aedes albopictus. Parasites and Vectors.

[CR36] Okogun GR, Anosike JC, Okere A, Nwoke B, Esekhegbe A (2003). Epidemiological implications of preferences of breeding sites of mosquito speciesin Midwestern Nigeria. Annals of Agricultural Environmental Medicine.

[CR37] Ortiz DI, Piche-Ovares M, Romero-Vega LM, Wagman J (2022). & Troyo, a..

[CR38] Patz, J. A., Olson, S. H., Uejio, C. K., & Gibbs, H. K. (2008). *Medical Clinics of North America, 92*(6), 1473–1491. 10.1016/j.mcna.2008.07.007. 10.1016/j.mcna.2008.07.007 19061763

[CR39] Pontius RG, Millones M (2011). Death to Kappa: Birth of quantity disagreement and allocation disagreement for accuracy assessment. International Journal of Remote Sensing.

[CR40] Pontius RG, Schneider LC (2001). Land-cover change model validation by an ROC method for the Ipswich watershed, Massachusetts, USA. Agriculture, Ecosystems Environment.

[CR41] Pontius RG, Boersma W, Castella J-C, Clarke K, de Nijs T, Dietzel C, Duan Z, Fotsing E, Goldstein N, Kok K (2008). Comparing the input, output, and validation maps for several models of land change. The Annals of Regional Science.

[CR42] Préau C, Isselin-Nondedeu F, Sellier Y, Bertrand R, Grandjean F (2019). Predicting suitable habitats of four range margin amphibians under climate and land-use changes in southwestern France. Regional Environmental Change.

[CR43] Price B, Kaim D, Szwagrzyk M, Ostapowicz K, Kolecka N, Schmatz DR, Wypych A, Kozak J (2017). Legacies, socio-economic and biophysical processes and drivers: The case of future forest cover expansion in the Polish Carpathians and Swiss Alps. Regional Environmental Change.

[CR44] Rakotoarinia, M. R., Blanchet, F. G., Gravel, D., Lapen, D. R., Leighton, P. A., Ogden, N. H., & Ludwig, A. (2022). Effects of land use and weather on the presence and abundance of mosquito-borne disease vectors in a urban and agricultural landscape in Eastern Ontario, Canada. . *PLoS One, 17*(3). 10.1371/journal.pone.0262376 10.1371/journal.pone.0262376 PMC8912203 35271575

[CR45] Reiskind, M., Griffin, R., Janairo, M., Hopperstad, K. J. M., & Entomology, V. (2017). Mosquitoes of field and forest: The scale of habitat segregation in a diverse mosquito assemblage. *31*(1), 44-54.10.1111/mve.12193 10.1111/mve.12193 27759165

[CR46] Reiter P (2001). Climate change and mosquito-borne disease. Environmental Health Perspectives.

[CR47] Roberts D, Irving-Bell R (1997). Salinity and microhabitat preferences in mosquito larvae from southern Oman. Journal of Arid Environments.

[CR48] Sakayarote K, Shrestha RP (2019). Simulating land use for protecting food crop areas in northeast Thailand using GIS and Dyna-CLUE. Journal of Geographical Sciences.

[CR49] Schrojenstein Lantman, J. V., Verburg, P. H., Bregt, A., & Geertman, S. (2011). Core principles and concepts in land-use modelling: A literature review. *Land-use Modelling in Planning Practice*, 35–57. 10.1007/978-94-007-1822-7_3

[CR50] Silver, J. B. (2007). *Mosquito ecology: Field sampling methods. Springer Science & Business Media*. Springer Science & Business Media. 10.1007/978-1-4020-6666-5

[CR51] Smith PG (2015). Long-term temporal trends in agri-environment and agricultural land use in Ontario, Canada: Transformation, transition and significance. Journal of Geography and Geology.

[CR52] Team, R. C. (2020). *R: A language and environment for statistical computing*. R Foundation for Statistical Computing, Vienna, Austria. Retrieved November 10, 2021 from https://www.R-project.org

[CR53] Tizora P, Le Roux A, Mans G, Cooper A (2018). Adapting the Dyna-CLUE model for simulating land use and land cover change in the Western Cape Province. South African Journal of Geomatics.

[CR54] Trisurat Y, Alkemade R, Verburg PH (2010). Projecting land-use change and its consequences for biodiversity in Northern Thailand. Environmental Management.

[CR55] Van der Sluis T, Pedroli B, Frederiksen P, Kristensen SB, Busck AG, Pavlis V, Cosor GL (2019). The impact of European landscape transitions on the provision of landscape services: An explorative study using six cases of rural land change. Landscape Ecology.

[CR56] Verburg PH, Overmars KP (2009). Combining top-down and bottom-up dynamics in land use modeling: Exploring the future of abandoned farmlands in Europe with the Dyna-CLUE model. Landscape Ecology.

[CR57] Verburg PH, Soepboer W, Veldkamp A, Limpiada R, Espaldon V, Mastura SS (2002). Modeling the spatial dynamics of regional land use: The CLUE-S model. Environmental Management.

[CR58] Verburg PH, de Nijs TC, van Eck JR, Visser H, de Jong K (2004). A method to analyse neighbourhood characteristics of land use patterns. Computers, Environment Urban Systems.

[CR59] Verburg, P. H., Veldkamp, T., & Lesschen, J. P. (2006). *Exercises for the CLUE-S model*. Retrieved February 2, 2022 from http://spinlab.vu.nl/wp-content/uploads/2016/09/ExerciseClues.pdf

[CR60] Verdonschot PF, Besse-Lototskaya AA (2014). Flight distance of mosquitoes (Culicidae): A metadata analysis to support the management of barrier zones around rewetted and newly constructed wetlands. Limnologica.

[CR61] Vynnycky, E., & White, R. (2010). *An introduction to infectious disease modelling*. New York: OUP Oxford.

[CR62] Waddell EH, Banin LF, Fleiss S, Hill JK, Hughes M, Jelling A, Yeong KL, Ola BB, Sailim AB, Tangah J (2020). Land-use change and propagule pressure promote plant invasions in tropical rainforest remnants. Landscape Ecology.

[CR63] Waiyasusri K, Wetchayont P (2020). Assessing long-term deforestation in nam san watershed, loei province, thailand using a dyna-clue model. Geography, Environment, Sustainability.

[CR64] Wang M, Sun X (2016). Potential impact of land use change on ecosystem services in China. J Environmental Monitoring Assessment.

[CR65] Xu L, Li Z, Song H, Yin H (2013). Land-use planning for urban sprawl based on the clue-s model: A case study of Guangzhou, China. Entropy.

[CR66] Zhang P, Liu Y, Pan Y, Yu Z (2013). Land use pattern optimization based on CLUE-S and SWAT models for agricultural non-point source pollution control. Mathematical Computer Modelling.

[CR67] Zhang L, Nan Z, Yu W, Ge Y (2016). Hydrological responses to land-use change scenarios under constant and changed climatic conditions. Environmental Management.

