# Supplementary material for: Future land-use change predictions using Dyna-Clue to support mosquito-borne disease risk assessment
Source: Environ Monit Assess. 2023 Jun 7;195(7):815. doi: 10.1007/s10661-023-11394-4 (PMC10246872; doi:10.1007/s10661-023-11394-4)
Supplement: Supplementary file 1 — Supplementary file1 (PDF 2993 KB) [file 10661_2023_11394_MOESM1_ESM.pdf]

Future land use change predictions using Dyna-Clue to support mosquito-borne disease risk assessment

Miarisoa Rindra Rakotoarinia[1,2], Ousmane Seidou[3], David R. Lapen[4], Patrick A. Leighton[1,2], Nicholas H. Ogden[2,5], Antoinette Ludwig[2,5]

[1] *Département de Pathologie et Microbiologie, Faculté de Médecine Vétérinaire, Université de Montréal, 3200 Sicotte, Saint-Hyacinthe, Québec, J2S 2M2, Canada*

[2] *Groupe de Recherche en Épidémiologie des Zoonoses et Santé Publique (GREZOSP), Faculté de Médecine Vétérinaire, Université de Montréal, 3200 Sicotte, Saint-Hyacinthe, Québec, J2S 2M2, Canada*

[3] *Department of Civil Engineering, University of Ottawa, 161 Louis Pasteur, Ottawa, Ontario, K1N 6N5, Canada*

[4] *Ottawa Research Development Centre, Agriculture and Agri-Food Canada, 960 Carling Ave, Ottawa, Ontario, K1A 0C6, Canada*

[5] *Public Health Sciences division, National Microbiology Laboratory, Public Health Agency of Canada, 3190 Sicotte, Saint-Hyacinthe, Québec, J2S 2M2, Canada*

Corresponding author: miarisoar@gmail.com

Supplemental Figure 1. Driving factors**:** (a) population density; (b) distance to water; (c) distance to cities, (d) distance to small cities, (e) distance to major cities, (f) distance to paved roads and (g) distance to major roads.

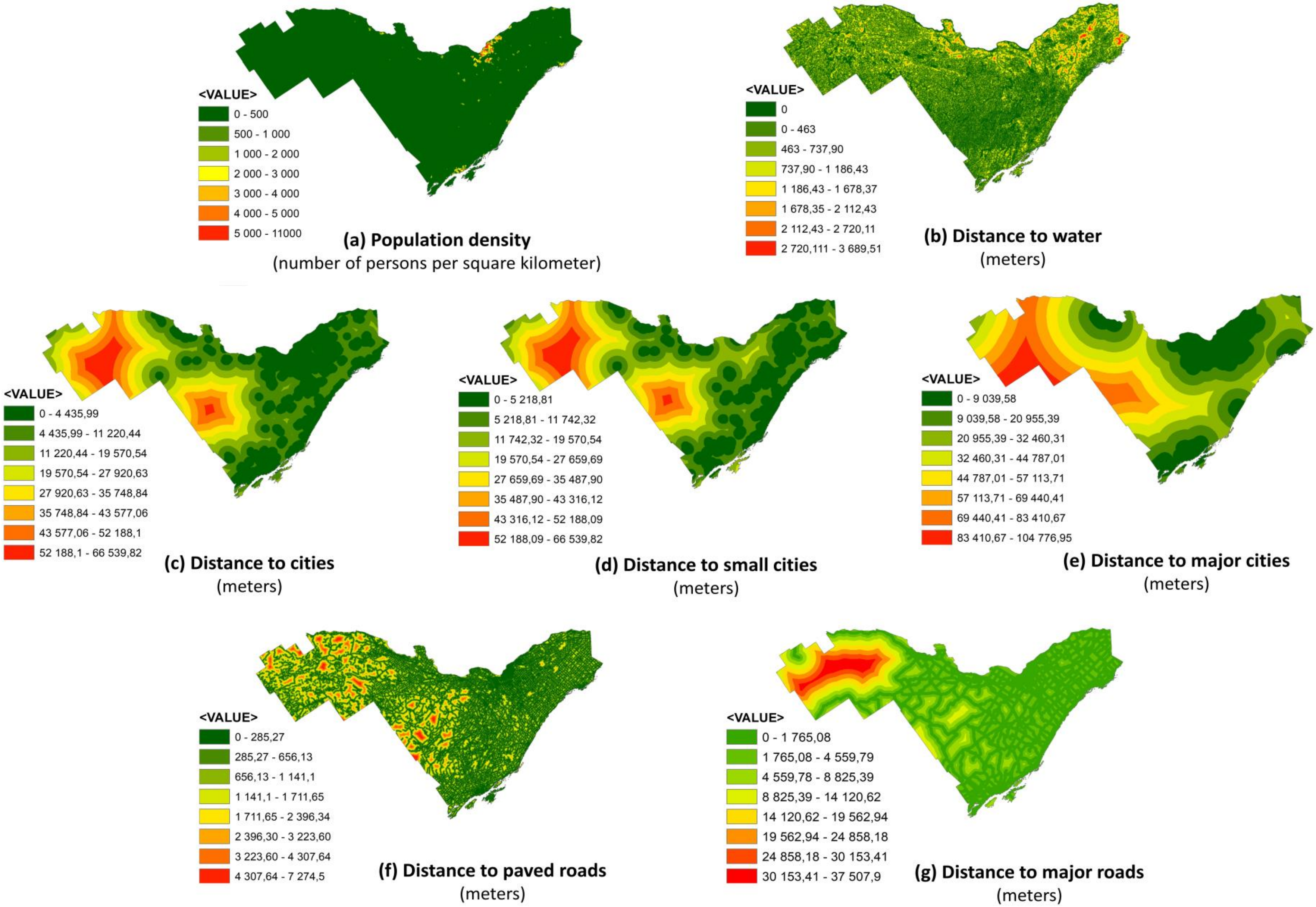

Supplemental Figure 2. Enlargement of the land-use patterns in 2030, 2050, and 2070 simulated by the Dyna-CLUE model in the (a) north western, (b) middle north western, (c) middle north eastern, (d) north eastern, (e) central and (f) and southern parts of the study area.

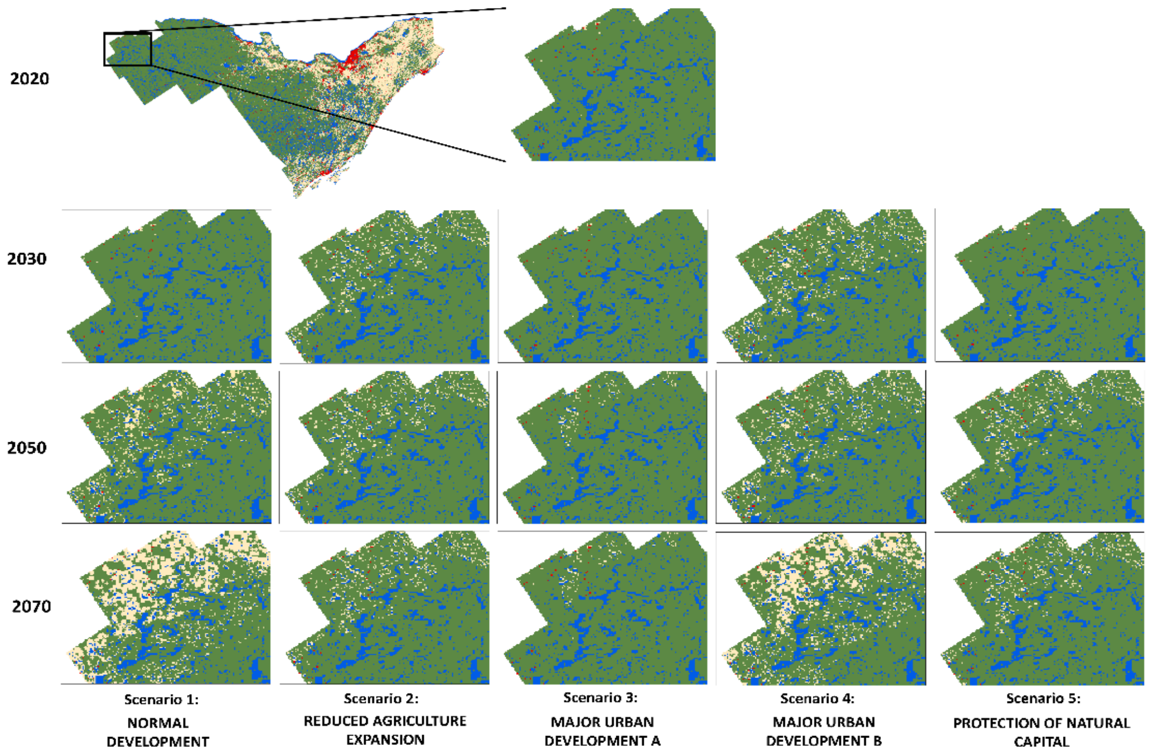

(a)

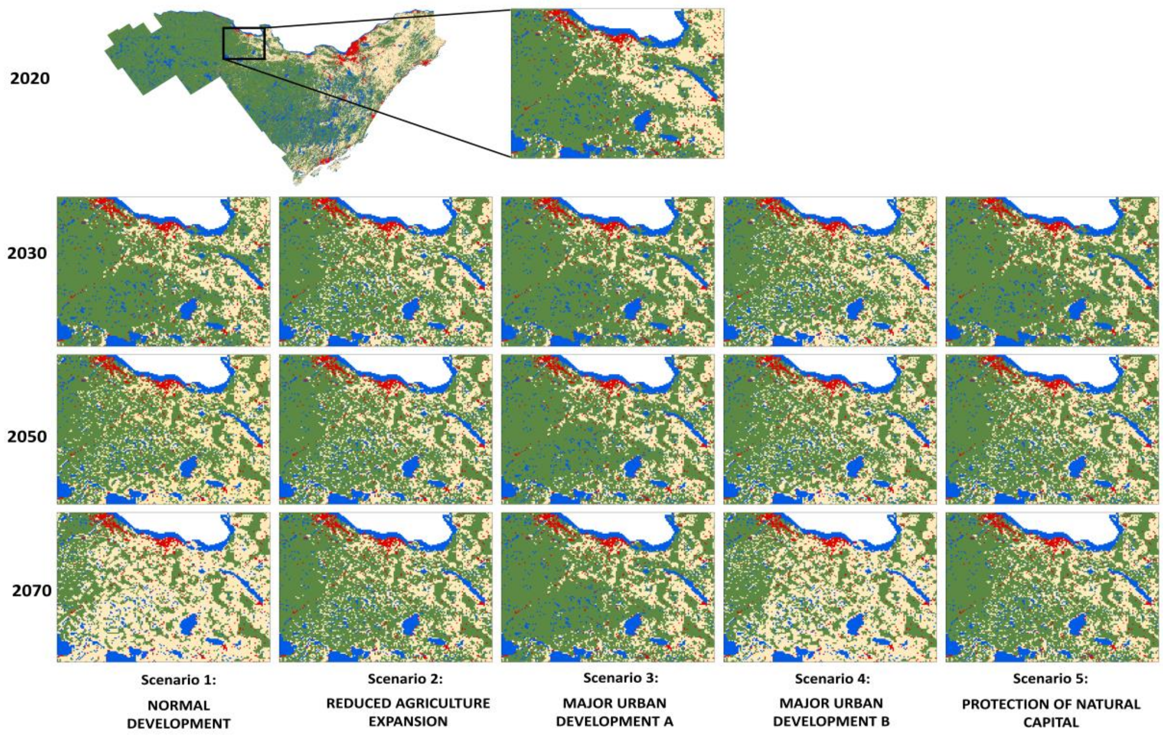

(b)

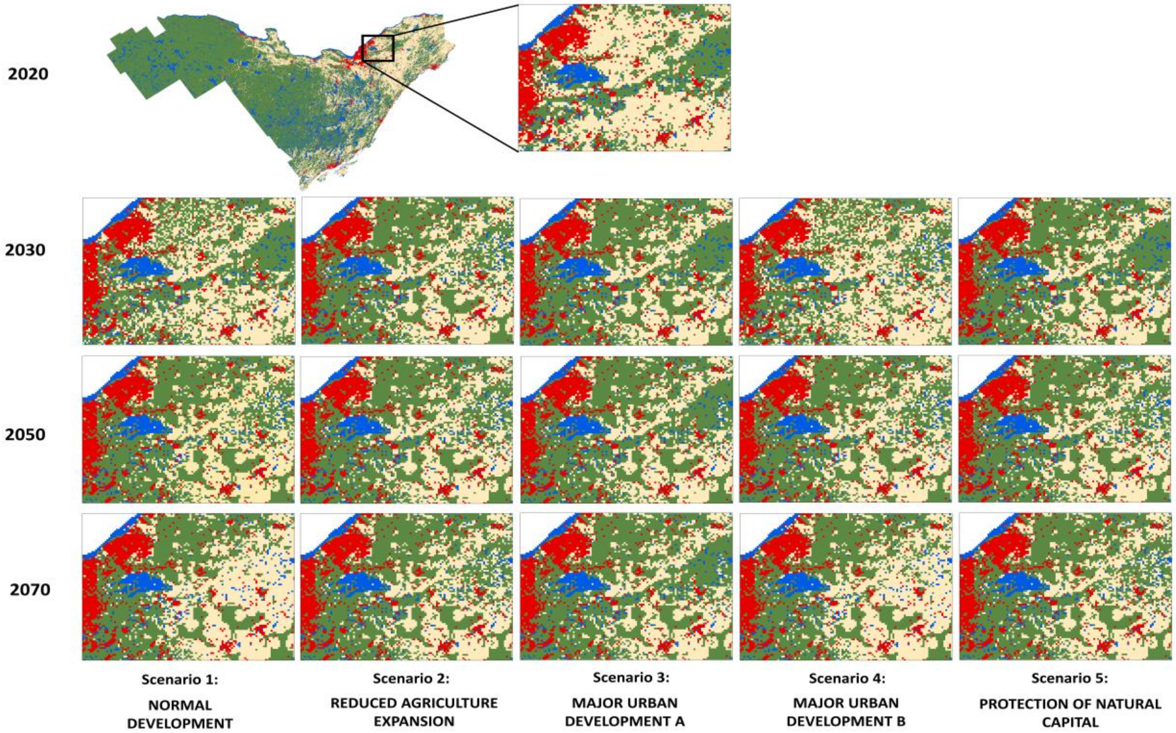
2020
2030
2050
2070
Scenario 1:
NORMAL DEVELOPMENT
Scenario 2:
REDUCED AGRICULTURE EXPANSION
Scenario 3:
MAJOR URBAN DEVELOPMENT A
Scenario 4:
MAJOR URBAN DEVELOPMENT B
Scenario 5:
PROTECTION OF NATURAL CAPITAL

(c)

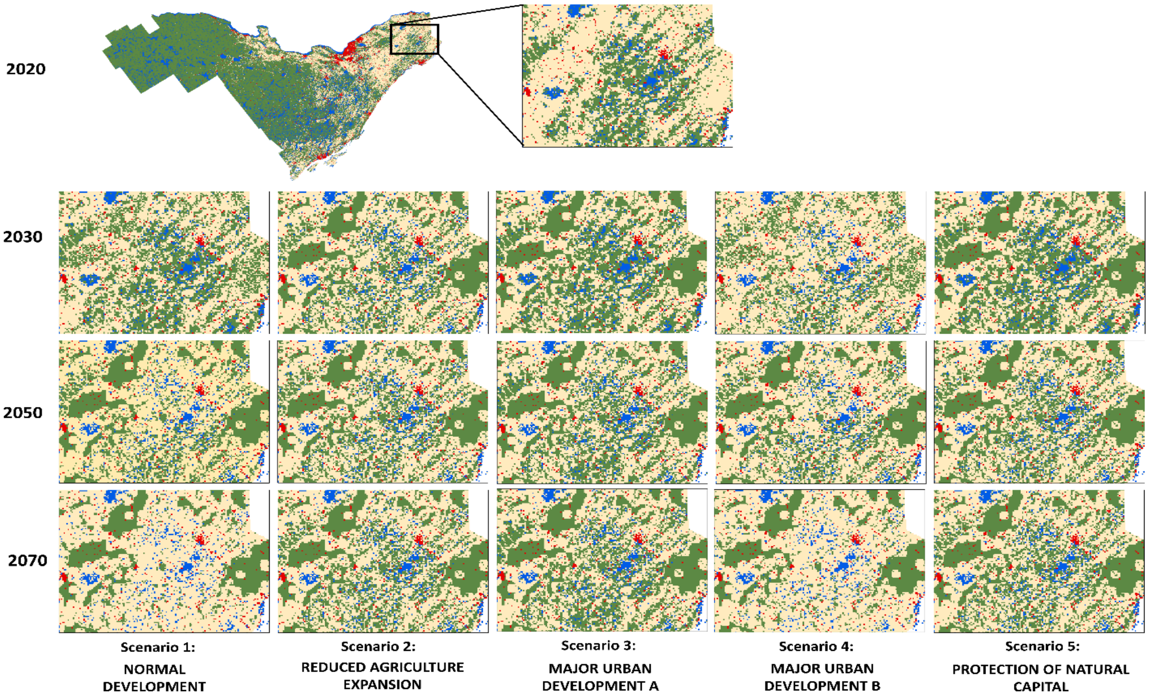
2020
2030
2050
2070
Scenario 1:
NORMAL DEVELOPMENT
Scenario 2:
REDUCED AGRICULTURE EXPANSION
Scenario 3:
MAJOR URBAN DEVELOPMENT A
Scenario 4:
MAJOR URBAN DEVELOPMENT B
Scenario 5:
PROTECTION OF NATURAL CAPITAL

(d)

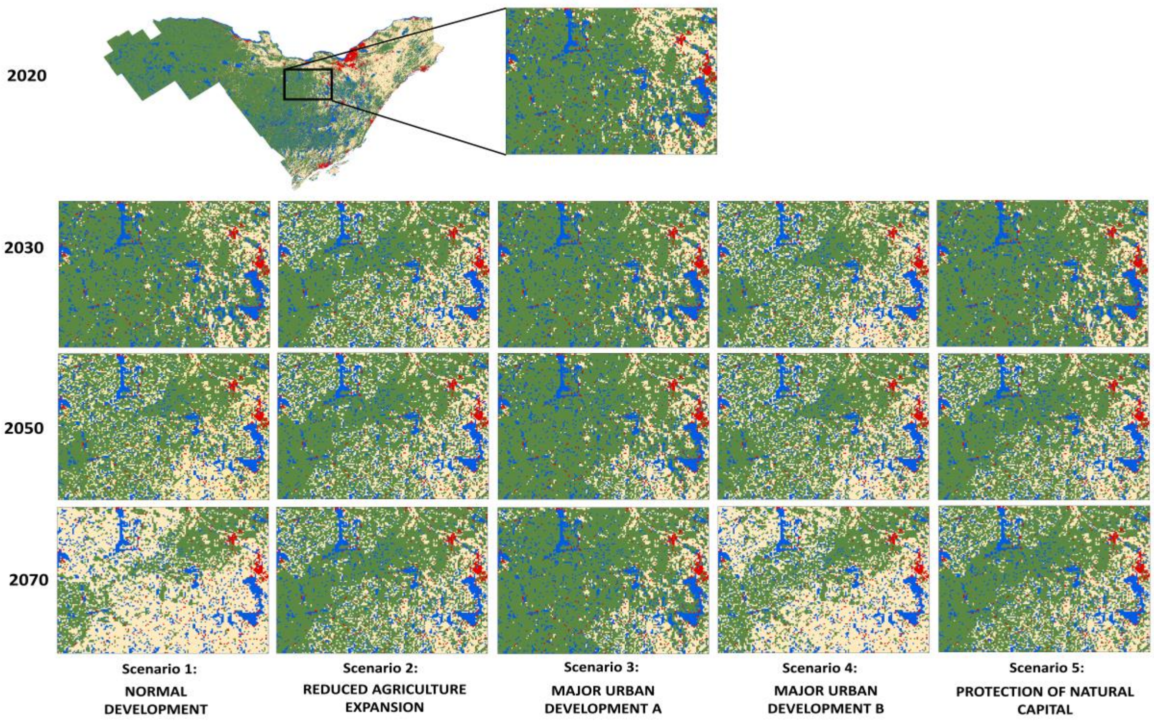

(e)

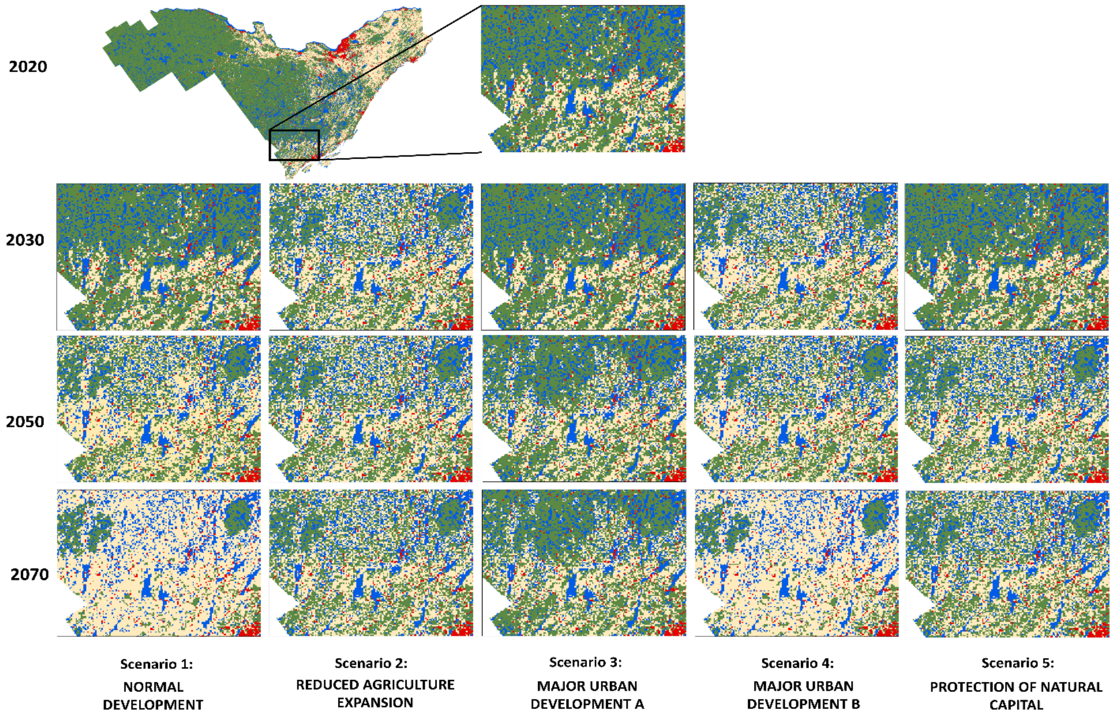

(f)

Note: These figures should be interpreted with caution because on a smaller scale, the results seem counter-intuitive. For example, more forested areas are found in scenario 3 (major urban development A) compared to scenario 5 (protection of natural capital). It is necessary to consider the results on a larger scale as presented in figure 7
